# The Facilitators and Barriers of Adopting Amylase-Rich Flour to Enhance Complementary Foods in the Kersa District Community of Eastern Ethiopia

**DOI:** 10.3390/nu13030838

**Published:** 2021-03-04

**Authors:** Asnake Ararsa Irenso, Shiferaw Letta, Addisu S. Chemeda, Abiyot Asfaw, Gudina Egata, Nega Assefa, Karen J. Campbell, Rachel Laws

**Affiliations:** 1 Institute for Physical Activity and Nutrition (IPAN), School of Exercise and Nutrition Science, Deakin University, 221 Burwood Highway, Burwood, VIC 3125, Australia; karen.campbell@deakin.edu.au (K.J.C.); r.laws@deakin.edu.au (R.L.); 2 School of Public Health, Haramaya University, P.O. Box 235 Harar, Ethiopia; 3 School of Nursing, Haramaya University, P.O. Box 235 Harar, Ethiopia; shife1973@gmail.com (S.L.); abinahom21@gmail.com (A.A.); negaassefa@yahoo.com (N.A.); 4 Department of Food Science and Postharvest Technology, Ambo University, P.O. Box 19 Ambo, Ethiopia; addisuus@gmail.com; 5 School of Public Health, Addis Ababa University, P.O. Box 9086 Addis Ababa, Ethiopia; gudina_egata@yahoo.com

**Keywords:** amylase-rich flour, germination, complementary food, Health Development Army, Ethiopia, child nutrition, undernutrition, malnutrition

## Abstract

Achieving the optimal transition to a family diet over the first two years of life has remained a challenge in Ethiopia. The use of amylase-rich flour (ARF) can improve complementary foods. However, utilisation requires an effective delivery strategy for upskilling the community to use ARF. The aim of this study was to explore facilitators and barriers of cascading ARF skills to improve complementary foods. The study was conducted in Gale Mirga kebele of Kersa district in Eastern Ethiopia in 2016. The study utilised exploratory qualitative research that used participatory action. Focus group discussions (FGDs) were conducted with the Health Development Army (HDA) leaders, religious leaders, and observation of participatory complementary food demonstrations. Cultural acceptability and the presence of HDA structure that supports skill development were identified as key facilitators to ARF use. On the other hand, the potential barriers to expanding ARF skill were lack of sustainability of external skill support for HDA leaders, perceived time constraints, unsuitable demonstration settings, cooking method, and large group size. The indigenous community’s knowledge of germination has not been used to improve complementary foods. The universal use of ARF requires integration into the Health Extension Programme (HEP) with support and supervision for HDA leaders.

## 1. Introduction

Achieving nutrient needs for healthy growth and development during the transition to a family diet over the first two years of life has remained a challenge in low-income settings such as Ethiopia [1]. According to the Ethiopian Demographic and Health Survey (EDHS), only 14% of children between 6 to 23 months meet the recommended diversified diet [2]. These children frequently consume foods made from fewer than four food groups, mainly local staples such as grains, roots, and tubers, followed by vitamin-A-rich fruits and vegetables, cheese, yoghurt, or other milk products [2]. Overall, about 69% of children do not eat vegetables or fruit [1], and while breastfeeding continues, only eight percent of children consume meat, fish, and poultry [2].

The poor dietary quality is attributed to low food availability, low family income, and associated inadequate food access [3,4,5,6,7], resulting in child malnutrition. According to the cost of hunger estimates for Ethiopia, child malnutrition causes 4.4 million annual additional morbidity episodes and 28% of under-five mortality in Ethiopia. About 44% of healthcare costs associated with undernutrition occur before the child turns one year old, which is partly attributed to the quality of complementary foods [8].

Complementary foods are foods and liquids given to infants, along with breast milk, when the nutritional requirements can no longer be met by breast milk alone [9]. Complementary foods can be industrially processed foods for mothers who can afford them. However, the nutrient fortified commercial infant foods are unavailable for most rural Ethiopian populations [10,11]. The second type is low-cost complementary food, which is prepared from locally available ingredients using commonly used household technologies suitable for resource-limited settings [12]. Locally processed complementary foods are the focus of this paper.

Some of the problems related to typical low-cost cereal- and pulse-based foods include their high water-holding properties (gelatinisation) during cooking, high viscosity and bulky consistency, and low nutrient and energy density that falls short of the child’s nutritional needs, especially for infants [13]. Another challenge of cereal–legume-based complementary foods is their high content of antinutrients such as phytates, lectins, tannins, and enzyme inhibitors, including protease and amylase inhibitors [14,15]. These antinutrients reduce the digestibility of foods and nutrient absorption. For instance, phytates bind to iron, calcium, zinc, reducing their bioavailability. The inhibition of calcium is associated with a lower digestibility rate of starch, and iron and zinc, increasing the risk of micronutrient deficiency [16].

Overall, homemade complementary foods have a risk of low diversity, low nutrient bioavailability, and caregivers have insufficient knowledge and skills to deal with these limitations [17]. Thus, meeting the child’s nutritional needs depends on accurate nutrition information and skilled support that caregivers get from the family, community, and healthcare system [12]. One viable option for improving homemade complementary foods is using simple household food processing methods such as germination [18].

Germination starts by selecting and cleaning whole grains, soaking the grains with an equal amount of water for 12 h, then draining and allowing them to germinate for 48 h at room temperature in an airtight plastic container in a cool and dark place. The germinated grains are then sundried by turning regularly, then roasted on a low flame until dry and milled into homogenous flour over four days [19,20]. The flour can be stored in a sealed container for a month [21].

Germinating grains increases the enzyme amylase [22]; hence, the flour is referred to as amylase-rich flour (ARF). ARF liquefies the thick starch of ungerminated flour [10,23] and facilitates digestion [24]. The amount of ARF needed to improve complementary foods is equivalent to 5% of a volume of prepared food, for example, 1 tsp (5 g) per 100 g of food [25]. The revised Codex Alimentarius Commission, the international food standards-setting body established by the FAO and the WHO, recommended using germinated cereal flour [26].

Intervention studies conducted in Bangladesh [7], India [27], Malawi [28], Tanzania [29], Congo [30], and Zambia [31] revealed the potential of ARF in increasing the energy and nutrient density of complementary foods when it was combined with the consumption of animal-source foods and nutrient supplementation. While ARF can narrow the nutrient gap, children are still likely to need more nutrients such as Vitamin A, iron, and calcium from other sources. Thus, ARF is only part of the solution to nutritional deficiencies [28,32]. Furthermore, using ARF is time-consuming and labour-intensive, and it has high costs associated with its frequent processing [19,20].

Improving child feeding practices requires practical messages that fit the cultural environment [33] with the right information for mothers and a skilled support system [17]. In line with the recommendation, Gibson suggested disseminating germination skills to improve complementary foods and the participatory research process to achieve high acceptability [34]. However, evaluation of community participation has been lacking in previous studies of ARF use in Ethiopia. For instance, studies on complementary foods based on sorghum [10,23] and fava beans [35], dry red beans, and chickpeas [36] were all laboratory-based. The focus of these studies was the determination of changes in nutrient and antinutrient contents, cooking time, and sensory evaluation of cooked foods with volunteer participants.

On the other hand, community-based studies related to germination have focused on testing community acceptability with mothers [37,38] through cooking demonstrations by Health Extension Workers (HEWs) [39]. These studies did not examine how best to upskill mothers and the broader community in the food processing skills required to produce ARF or the opportunities and challenges of scaling up skill development in the community using existing peer support networks.

A viable option for scaling up ARF skill development might be using the Health Development Army (HDA), a network of 25 to 30 women, organised for community mobilisation and adoption of improved health practices in Ethiopia. However, the potential of HDA as a channel for ARF skill development is yet to be explored. Hence, this study aims to explore the facilitators and barriers of using the HDA as an avenue for upskilling the community to using ARF as a component of complementary foods.

## 2. Materials and Methods

### 2.1. Study Setting 

The study was conducted from March to September 2016 in Gale Mirga kebele, one of the 38 kebeles of Kersa district of Eastern Ethiopia. The district is characterised by high child crude birth (37.2 per 1000 population) and death rates (7.8 per 1000). Furthermore, the district has one of the highest infant (46.9 per 1000 live births), and under-five (77.4 per 1000 live births) mortality rates in Ethiopia, partly attributed to child undernutrition. The common food crops produced in the district include wheat, barley, sorghum, maise, potatoes, and vegetables [40]. 

### 2.2. Study Design 

**Figure 1:**
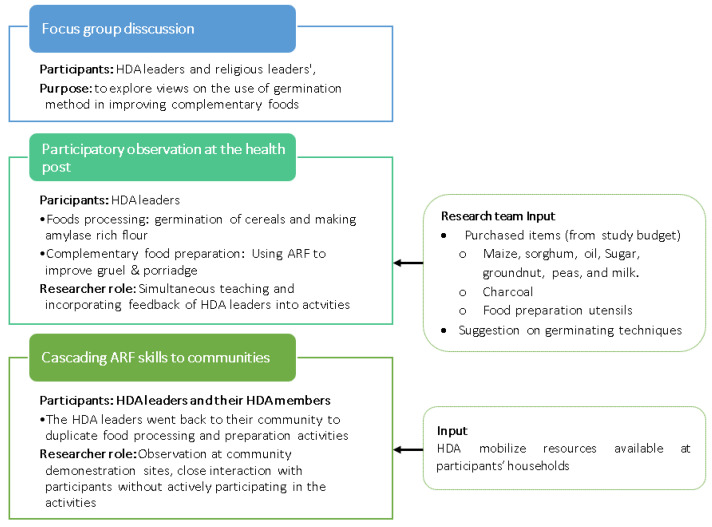
Illustration of the method used and activities involved in the study. HDA, Health Development Army.

The transformative research paradigm guided the study. The rationale for choosing this paradigm was the overall intentions of the research of enhancing the complementary foods by promoting and scaling up simple household level technology, the germination, via active community participation. In line with this paradigm, participatory action research informed the study design [41,42]. The study was exploratory qualitative research that used focus group discussions (FGDs) involving HDA leaders and religious leaders separately and participant observation with HDA leaders and their group members. The FGD was conducted with religious leaders to inform our understanding of the religious implication of using germinated cereal flour. Likewise, the FGDs with HDA leaders were intended to examine experiences of ARF use in complementary foods in their communities/groups. The participant observation was used to examine the natural settings and interactions of a typical HDA-led complementary food demonstration (Figure 1).

### 2.3. Sampling Strategy and Data Collection Methods

#### 2.3.1. Focus Group Discussion 

Separate FGDs were conducted with HDA and religious leaders. Gale Mirga kebele has 23 HDA leaders, with five leaders having children between 6 to 23 months of age. In addition, seven religious leaders were identified (both Muslim and Christian). All HDA leaders and religious leaders were invited verbally and showed a willingness to participate in the FGDs (Figure 1).

The HDA is a pre-existing women’s group formed by the government, and it is organised in two tiers. The larger group consists of a network of 25 to 30 women, led by a woman (HDA) leader who is a grassroots volunteer delivering health messages to households. The smaller groups of five women with one leader (popularly called one-to-five women’s networks) are organised based on their household proximity to facilitate the adoption of improved health practices [43]. HDA leaders work with and are supported by Health Extension Workers (HEWs) to deliver health promotion activities [43]. Both tiers of HDA leaders were involved in the study.

HDA leaders were approached for their potential to serve as a vehicle for promoting the villagers’ skills needed to produce and use ARF in improving complementary foods. According to evidence from the kebele health post, there were 23 HDA leaders in the kebele. Since this number is manageable, all HDA leaders were invited verbally, and all agreed to participate in three FGDs held with these leaders. 

Two investigators moderated the FGDs, one for facilitating the discussion, the other for taking notes. The key topics discussed with religious and HDA leaders were their views on using germinated products in child foods. The use of a pre-existing HDA group created a relaxed environment for the FGDs. The FGDs were conducted at a local primary school, and discussions were audio-recorded with the participants’ permission.

#### 2.3.2. Participatory Complementary Food Demonstration

The health post and the community’s observational data were captured using a checklist that primarily covered food processing and preparation, group size and interaction, and acceptability of the foods and demonstration settings. The observation was supplemented with field notes and photographs without identifying participants’ identities (Figure 1). The two phases of participant observation conducted at the health post and in the communities are presented as follows. 

#### 2.3.3. Participant Observation at the Health Post

The same HDA leaders involved in the FGD were also approached to participate in participatory complementary food demonstrations. HDA leaders were primarily responsible for disseminating nutrition messages through home visits and their group meetings. The 23 HDA leaders were divided into four groups, each with four to six members, formed based on their household proximity. Three food processing and preparation sessions were provided for each group.

The research team was fully engaged with the HDA leaders to show them how to process ARF and use it to prepare complementary foods and incorporated their feedback, such as ingredients available in their village and the time that works for participants, into activities. The health post activities commenced with purchasing the necessary ingredients and cooking utensils from the local market based on the HDA leaders’ suggestion and the research team’s financial contribution (Figure 1).

Each of the four HDA leader teams prepared amylase-rich flour (ARF) from one of the following cereal types: wheat, maise, white and red sorghum. The demonstrations involved simultaneous training, receiving, and incorporating participants’ feedback, such as a schedule that works for participants and sharing experiences among participants.

The HDA leader groups met at the health post three times during the ARF processing. The first meeting was before starting the germination process for overall orientation on germinating cereals, group formation, and taking necessary inputs used for activities in their villages. The second meeting was after the cereals were germinated, and the four groups brought the germinated cereal and showed it to other HDA groups. The third meeting was after the germinated cereals were dried and ready for milling. In addition to ARF, each group prepared the regularly promoted multi mix complementary flour used along with ARF in the ratio of 3:1 for staple foods to nuts/legumes made of similar ingredients [26].

Three sessions of complementary food preparation were conducted at the health post. The HDA leaders prepared porridge from regular flour (the ungerminated flour) and added half a spoon of germinated flour or extra if they need thinner porridge consistencies. In the first round of demonstration, the HDA leaders shared the prepared porridge among themselves. In the subsequent two meetings, five HDA leaders who had children aged six months to two years were allowed to bring them to the demonstration place to share the food. A checklist was used to assess the acceptability of the food judged by the mothers’ reactions and comments during group interaction and the amount (presence of leftovers) consumed by children serving the food.

#### 2.3.4. Cascading ARF Skills to Communities 

After the initial demonstration to HDA leaders at the health post, each HDA leader went back to their group to repeat the complementary food processing and preparation demonstration in three sessions divided into two parts. In the first two sessions, the HDA leaders mobilised their members to produce ARF, and the third scheduled meeting was a cooking session observed by the research team. Thus, a total of 23 demonstrations were scheduled. This time they mobilised their community members and pooled resources needed to duplicate experiences shared at the health post (Figure 1). The decision about when to conduct observations was arranged with study participants in coordination with the HDA leaders that organise community meetings.

During the health post activities, the researchers had an active observer role of providing inputs needed for activities, instructing HDA leaders on making ARF and its use in complementary foods and close interaction with members (Figure 1) via receiving their feedback to be used in the activities.

On the other hand, observations of cascading activities were limited to interaction and activities among the study participants. The practical support provided for food processing and preparation activities was up to the HDA leaders. Three investigators, who were proficient speakers, readers, and writers of the native language, Afan Oromo, participated in the observation. During the observation process, the investigators interacted with participants to take notes on their views, took pictures of the demonstration settings, and examined the overall group interaction but did not provide any specific instructions or advice.

### 2.4. Data Processing and Analysis

#### 2.4.1. The FGD

The audio recordings were transcribed and translated to English by the research team. The draft transcript was checked for authenticity and accuracy, then imported and coded using ATLAS.ti 7. Text segments were described in detail by attaching appropriate codes (the initial coding frameworks merged under subthemes by the lead author with other researchers and analysed inductively based on the checklists). Finally, related subthemes were merged into a major theme, triangulated with representative identified excerpts.

#### 2.4.2. Participatory Observations

Observation notes, notes on participants’ views that supported the observations, and photographs were imported and coded using ATLAS.ti 7. The analysis was based on the observation checklist and was thus a deductive analysis. Finally, the observation findings were presented by triangulating them with an FGD.

## 3. Results

We conducted three FGDs with HDAs (*n* = 23 leaders) during the first phase of fieldwork and one FGD with religious leaders (*n* = 7). While all the HDA participants were women (some had infants and young children), all religious leaders were males. Three food processing and preparation sessions were provided for HDA leaders at the health post, and then each HDA group went back to their community and redemonstrated the activities by pooling resources. The analysis revealed a range of facilitators and barriers to the use of ARF as follows.

### 3.1. Facilitators of the Use of Germinated Flour

#### 3.1.1. Cultural Acceptability

According to HDA leaders, the community was aware of household food processing methods such as germination, but not its application to the enhancement of complementary foods. Both religious leaders and HDA leaders associated germination with local alcohol production, which is unacceptable in the rural Muslim communities. However, the FGD with religious leaders revealed that germination is acceptable if the purposes are improving infant and young child feeding. HDA leaders also reinforced this idea. One HDA leader said:

*“We have never used, or no one showed us how to enhance the complementary foods using germination. Concerning the cultural and religious implication of the germination, as far as it is for a child benefit (not alcohol), it is okay. However, we have never been introduced to these processing methods before.”*

**Figure 2:**
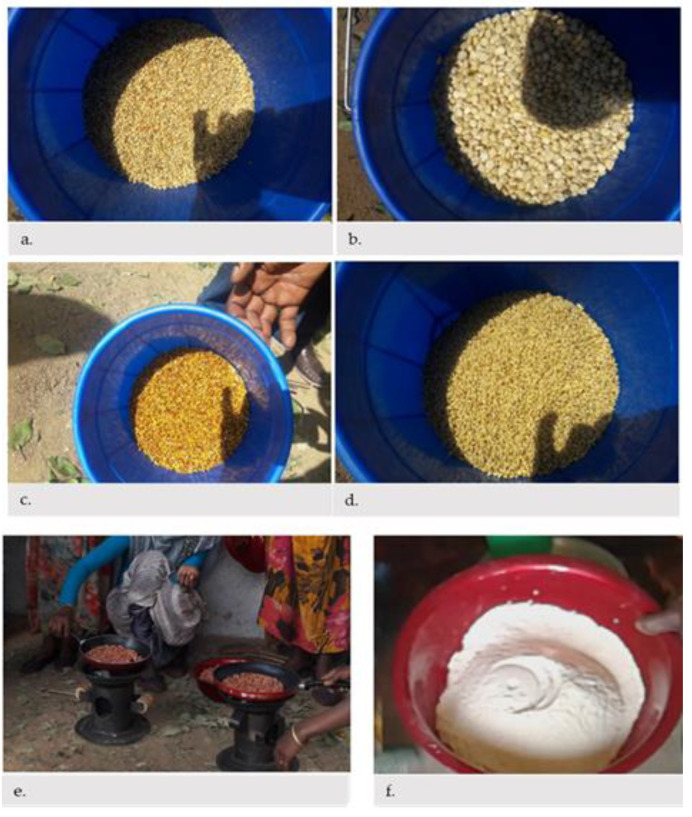
Types of food groups used to make multi mix and germinated flour. (**a**). Germinated white sorghum; (**b**). Germinated maize; (**c**). Germinated red sorghum; (**d**). Germinated wheat; (**e**). Ground nut; (**f**). Ungerminated multi mix flour.

HDA leaders were eager to learn how to germinate and use germinating grain flour. During the demonstration, they described the porridge they prepared with ARF of the right consistency, taste, and appearance for the child (Figure 2).

#### 3.1.2. Taste Acceptability 

**Figure 3:**
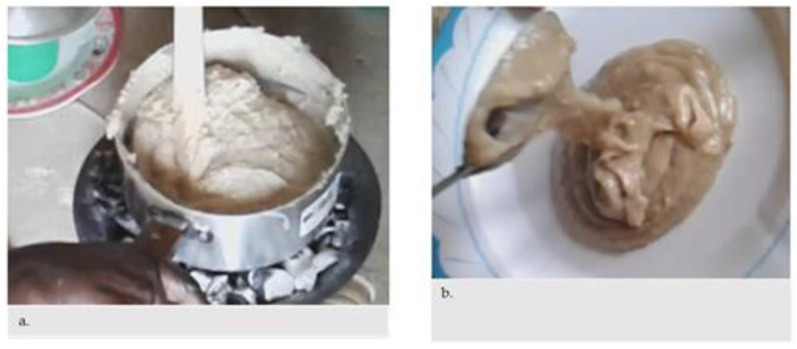
(**a**). Porridge of ungerminated multi mix; (**b**). Same porridge enriched with germinated flour.

Compared to the multi mix ungerminated flour, the participants confirmed that the porridge that contained ARF had a softer consistency without adding extra water (Figure 3). Using child appetite as a guide to the adequate amount of food, the children ate the food in one sitting. One HDA leader that brought her child to the demonstration said:

*“I have never used germinated cereals before. Porridge and gruel made by adding germinated flour are good because its taste and appearance are good. Furthermore, it adds softness to baby food, and it’s easy to swallow. The thinner consistency is good. I used to add water to thin baby foods. I think it provides a better and balanced nutrient. I have tested it myself, and it did not stick to my throat.”*

#### 3.1.3. Presence of HDA Structure and Sustainability of ARF Use

Repeated field observation showed that the HDA leaders had mobilised the community to duplicate the complementary food demonstration in their respective groups. However, there were variations across groups in terms of how the community members contributed to staples, wood, cooking utensils, and water and the time taken to process the grains and make them into flour. 

HDA leaders reported that group members who contributed mostly attended the meetings and actively participated in the demonstrations. In this regard, community mobilisation can be considered an opportunity to scale up ARF use skills in the communities. However, a range of barriers was identified for expanding the ARF use via HDA leaders. 

### 3.2. Barriers to Cascading ARF Use in the Community

#### 3.2.1. Lack of Sustainable Skill Support by Professionals

The HEWs are the workforce that supervises HDA leaders and supports their skills, but they have not been trained on ARF-related skills. Also, the HDA leaders will not have access to continuous support from the researchers A key area of focus for HEWs is malnutrition case detection and management, which accounts for a large proportion of their workload. This was identified as a barrier to expand and maintain malnutrition prevention and promotion activities such as ARF-related skills of HDA leaders. In this regard, empowering HEWs with ARF-related skills to enable regular supervisory support for HDA leaders could facilitate the ongoing skill transfer to households. The FGD3 HDA leader said:

*“Community health workers come to our village to identify a child with nutritional deficiencies rather than prevention of child undernutrition through demonstrating such a new complementary food preparation. Young mothers usually share the experience of their neighbours and older women. If the government shows us how to make good child foods not only for the child with deficiency but healthy baby, we can teach the mothers. Mothers follow HDAs leaders’ advice.”*

During the demonstration of ARF production to HDA leaders, the recurrent issue HDA leaders raised was the sustainability of the support-related skill required to make and use ARF. HDA leaders agreed that the Health Extension Workers (HEWs) are busy with multiple activities. As a result, mothers prefer HDA leaders over the HEWs to disseminate messages and skills related to feeding practices of infants and young children. An HDA leader explained:

*“HEWs did not usually engage in teaching community about the processing of baby foods the way HDA leaders did. A Health Development Army leader delivers whatever she has trained during a frequent public meeting and looks over her members regularly. We have mobile phone access, and mothers can call us when they need help. I think similar demonstrations should continue.”*

The roles and relationship between the HDA leaders and HEWs are well delineated. HDA leaders’ motivation as a frontline vehicle to promote nutrition messages reinforces the importance of delegating them to community nutrition activities such as the promotion of ARF. 

#### 3.2.2. Perceived Time Constraints Related to ARF Processing

While HDA leaders were enthusiastic about the ARF and confident in teaching community members how to make and use ARF, they were concerned about whether all mothers have the time to prepare two different flours. Most HDA leaders agreed that commercial production of ARF is a more viable option than preparing it at home. An HDA participant in the demonstration said:

*“I think we can prepare baby foods the way we have [been] trained. However, I cannot prepare ARF and give the baby year-round. Our cultivation, such as sorghum, takes a long time to harvest. Therefore, we must buy it from the market. I had better buy germinated flour from shops like a salt. Instead of buying other biscuits, we could spend the money on ARF.”*

#### 3.2.3. Unsuitable Demonstration Settings

**Figure 4:**
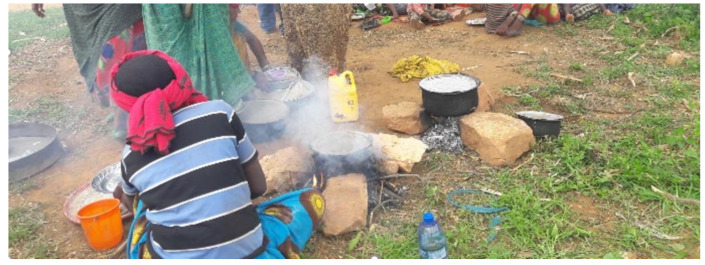
HDA leaders in complementary food preparation.

The demonstration settings were a vital consideration influencing mothers’ likely success in the ARF lessons. In this study, the demonstration HDA leaders organised for their communities was under the shade of a tree. This was unsuitable due to the uncomfortable sitting arrangements for participants (Figure 4). Furthermore, participants were continuously distracted from the demonstration by passers-by, who chatted with participants. There was also a water accessibility issue, with insufficient water available to maintain hygiene and ensure that prepared food was safe for consumption (Figure 4). 

#### 3.2.4. Cooking Method and Lengthy Preparation Time

During the demonstration sessions HDA leaders organised for their communities, women used an open wood three-stone fire on the ground to cook the complementary foods. This prolonged cooking time, and the smoke acted as a distraction. The communities routinely use the same method at home, but households modified the cooking methods, using a stove that contains a combustion chamber that conducts the heat to the cooker and with a chimney to reduce heat loss and cooking time. Nevertheless, the method is a barrier to scheduling regular complementary food demonstrations and attendants at the community settings. 

#### 3.2.5. Variability of Effective Group Size

The number of women that actively participated in the ARF demonstration was variable across the 23 HDA teams. An influential HDA leader had managed to pool resources and convene most of her group members, more than 15 in total. In comparison, most HDA leaders convened 8 to 12 members. However, due to the large group sizes, not all participants could actively participate in the demonstration. On the other hand, three HDA leaders managed to demonstrate with smaller group sizes of around six participants, resulting in better group member participation than the larger group. One HDA leader was unable to organise the meeting at all due to the inability to pool resources and organise a community meeting. 

## 4. Discussion

This is the first study to explore the facilitators and barriers of cascading community skill development for germinating cereals enhancing locally made complementary foods. The cultural and taste acceptability and the presence of the HDA structure were found to promote ARF use. On the other hand, the lack of sustainability of ARF skill support, perceived time constraints, unsuitable demonstration and cooking methods, and variability of the effective demonstration group size were potential barriers to the expanded use of ARF. Germination is an indigenous food processing method [44] used in Ethiopia [45] but with a low rate of use for complementary foods. This holds for our study and Southern Ethiopia [46]. Key to its acceptability, religious leaders and HDA leaders favoured germinated grain flour to improve complementary foods. Germinated seeds are used as ingredients to brew alcohol in Ethiopia [47,48]. This might lead to religious sensitivity and resistance to using products, and working with religious leaders might warrant use in similar environments to our study setting.

The approval of HDA and religious leaders around the use of germinated grain use has critical practical implications. The views of these leaders define emic knowledge, which are indigenous views reflecting themselves and their community [49]. Their affirmative views suggest that by embedding the implementation of germinated flour into the local context, the ARF use will have less resistance from the community’s insiders entitled to the final say. Hence, future studies should investigate if approval by these key instructors and integration of ARF into the nutrition component of Health Extension Programmes and the trained HEWs supporting HDA leaders and households are helpful to achieving a higher rate of ARF use.

There is variability in the awareness and use of germination in complementary foods across studies. For instance, our study participants had never used germination to enhance complementary foods. Little more than a third of Southern Ethiopian mothers practice pulse germination for complementary foods [50], and in Malawi, only four percent of mothers added germinated flour to complementary foods [51]. These findings consistently showed a low level of translation of germination into improving complementary foods across settings.

Low levels and variabilities in the application of germination in our study and those based in Southern Ethiopia, Nigeria, and Malawi [50,51,52] might be attributed to cross-cultural differences. In our view, describing and addressing these differences represents a first step [53] that policymakers should consider and learn from before adopting the best experiences around the use of germinated flour across contexts. 

On top of understanding the cultural context, successful expansion of ARF skills requires an appropriate implementation platform [53]. In line with a study conducted in Southern Ethiopia that delivered pulse sprouting education through women’s development team leaders, our study used a similar structure [46]. However, Hailu et al. [46] relied on self-reported use of pulse germination, and it was not verified with observation [46]. The findings of this study showed that the HDA leaders cascaded their ARF skill to available members, which they were supposed to deliver through the one-to-five networks, a structure key to adopting the improved practices [43]. The use of the HDA one-to-five networks ensures an appropriate group size to allow active participation and skill development. The use of the one-to-five networks should be reinforced in HDA leaders for nutrition education. 

Our study also revealed participant preference of HDA leaders over HEWs as a vehicle for promoting the use of germinated flour. A study conducted in Southern Ethiopia implemented a package of interventions, including using germinated pulse, via the HEWs at the mothers’ homes fortnightly for six months. The intervention helped most women use pulse germination for complementary foods. However, the study did not establish the feasibility and sustainability of frequent home visits and experiences of HEWs over the course of implementation [50]. 

Despite the barriers of ARF implementation via HDA leaders, Sako et al. [54] have reported mothers’ preferences for receiving nutrition information from HDA leaders over HEWs who might not have time to support them [54]. This is in line with the findings of this study. The choice between the HEWs and the HDA leaders might be seen in the light of task shifting, a process of delegating activities to less technical people, which is an increasingly practical option to reduce health system cost and efficacy [55]. At this juncture, to supplement the decision-making process, future studies should examine the cost saved and the efficacy of shifting complementary feeding activities to HDA leaders.

The productivity of community health workers such as HEWs and HDA leaders depends on knowledge and skills, motivation, and their work environment (supportive supervision and workload) [56]. In this regard, unlike Mulualem et al. [50] and other studies [20,21,29,30,46,50,51] that examined ARF use in complementary foods using existing health delivery platforms, our study has reported opportunities and challenges surrounding ARF implementation from HDA leaders’ perspectives. 

HDA leaders perceived that a key barrier for mothers might be the time required to prepare ARF, which is in accordance with the findings of Irenso et al. [57] where maternal time poverty was a crucial driver of suboptimal child feeding practices. Hence, the use of commercial ARF might be a good sustainable alternative. The preference and sustainability of using commercial ARF mentioned by participants, details of which are not currently available, over making it at home should be examined in future studies. The development of complementary commercial foods produced from locally sourced ingredients is one of Ethiopia’s research priorities. Future studies should examine the local use of commercial ARF in Ethiopia as a supplementary food for malnourished children and the general communities [58]. 

The barriers to the implementation of ARF, such as the use of fuel for cooking and access to adequate water needed to ensure food safety, can negatively influence ARF use. The problems are common to rural Ethiopian communities [2]. Findings from Uganda showed that the acceptability of improving complementary feeding practices depends on the ease of cooking, shorter cooking time, and use of local ingredients, which might hold for Ethiopia [59]. Hence, barriers to implementing ARF skills through participatory demonstration in the community call for multisectoral collaboration to improve the cooking methods with efficient fuel stoves with less smoke and arranging modest demonstration settings with water. 

The participatory approach is influenced by contextual factors such as group composition, dynamics, and size. These factors determine what it takes to mobilise the community, including resource pooling, sharing individuals’ experiences, and sustaining the interventions [43,58]. In these regards, our findings showed that considering the two-tier HDA system, the larger the group size, the better the resource pooling, but the less active the participation of group members, the factor that needs consideration in designing future interventions. Cascading skills to a smaller group size of a one-to-five network can foster active participation and retention of behaviours. Once the women internalise the skill and adopt the behaviour, they can prepare in a team with peers (other mothers) or individually. 

Finally, this study has a number of strengths and weaknesses. Strengths include the examination of ARF use in the typical rural setting using participatory approaches, observations of the opportunities and barriers of implementing ARF encountered by HDA leaders, and the recognition and use of religious leaders’ views. The study’s weakness was that we did not include other stakeholders’ views, such as HEWs and district health officers. Furthermore, the participatory approach used examined neither the effectiveness of ARF nor the rigorous testing of the acceptability and adoption of ARF by the mothers and other stakeholders. Hence, future research should examine a broader implementation of ARF with the involvement of relevant stakeholders and the longer-term production and use of ARF in the community following skill development and the subsequent impact on child feeding practices. Furthermore, we recommend a rigorous assessment of differences in maternal/infant and young children’s acceptability of complementary foods containing ARF versus those that do not contain ARF.

## 5. Conclusions

In conclusion, this study highlights the presence of awareness of germination as a food processing method that has not been translated into action to improve complementary foods. Despite the infrequent use, ARF was acceptable, and HDA structure was a readily available platform for implementing skills around ARF use. The HDA platform can be used to make ARF commercially available in the form of local women’s cooperatives, where women pool their resources to conduct business and consistently and reliably control the price of the products.

The universal ARF use requires integration into the nutrition component of the Health Extension Programme (HEP) so that HEWs can receive training on ARF skills, then support and supervise HDA leaders to upskill the community. Furthermore, government, communities, and other stakeholders can set minimum standards for participatory community demonstrations in terms of water access, energy sources, and cooking methods and the group size for demonstration. 

Concerning generalisability, the lessons learnt in this study may apply to similar resource-limited settings, although further research is required to explore any context-specific differences such as culture, health system structures, and existing policies. Future research should rigorously examine the implication of integrating ARF into routine nutrition activities on the potential use of ARF at home and its effectiveness in promoting child nutrition.

## Data Availability

The data that support the findings of this study are available with the principal investigator upon request.
